# Industrial Poisoning from Celluloid Varnish in the Aero-plane Industry

**Published:** 1916-03

**Authors:** 


### ABSTRACTS.

---

Have you ever felt that this department of the BUFFALO MEDICAL JOURNAL was lacking in abstracts of your specialty, or that such as were published lacked the expert judgement which you yourself could have exercised in selection and preparation? If not, you are more charitable than the editor has been to himself. If so, will you assist in abstracting from a few journals, and thus make this department more representative and more valuable? Incidentally, there are few forms of study more helpful to the worker than this.

---

**Industrial Poisoning from Celluloid Varnish in the Aeroplane Industry.** Koelsch, Muench. Med. Woch., Nov. 16, 1915. The danger lies in the solvents: chloroform, carbon tetrachlorid, acetone, amyl acetate, formic acid acetylene tetrachlorid, etc. The symptoms obviously differ but may be grouped as gastro-enteric with jaundice and enlargement of liver and anaemia ultimately, or as nervous, with headaches, paraesthesiae, tumor, paresis, etc. 14 cases are reported, two fatal. 9 cases of acetylene tetrachlorid are also reported specifically.

